# Detection of vanA genes in vancomycin-susceptible Enterococcus faecium isolates: implications for additional testing

**DOI:** 10.1099/acmi.0.000959.v4

**Published:** 2025-04-01

**Authors:** Victoria Jordan, Hemalatha Varadhan

**Affiliations:** 1 Department of Microbiology, NSW Health Pathology, John Hunter Hospital, New Lambton Heights, NSW 2305, Australia; 2 School of Medicine and Public Health, University of Newcastle, Callaghan, NSW 2308, Australia

**Keywords:** vancomycin-variable *Enterococci*, vancomycin-resistance *Enterococci*, *vanA*, *vanB*

## Abstract

To assess the frequency of silent vancomycin resistance, phenotypically susceptible *Enterococcus faecium* isolates underwent genotypic testing using Cepheid’s Xpert *vanA*/*vanB* PCR. A total of 6% of isolates had silent *vanA* genes. However, the clinical relevance of silent *van* genes and the lack of rapid, random-access genotypic methods poses an ongoing challenge to laboratories.

## Data Summary

The authors confirm that all supporting data, code and protocols have been provided within the article.

## Introduction

Vancomycin-resistant *Enterococci* (VRE) are a common cause of healthcare-associated infections and associated with significant morbidity and mortality, as well as limited treatment options and significant healthcare costs [1]. Acquired vancomycin resistance in *Enterococcus sp*., most frequently affecting *Enterococcus faecium,* is commonly due to acquisition of *vanA* or *vanB* genes [1]. VRE can be easily spread by contact with contaminated surfaces and objects; in addition, plasmid-mediated transfer of *van* genes to other organisms is possible [2]. Accurate identification of VRE, allowing timely infection control strategies and treatment where necessary, is, therefore, of significant clinical importance. Routinely, laboratories perform phenotypic antibiotic susceptibility testing, for example, automated broth microdilution instruments such as VITEK2 (BioMerieux, France) or BD Phoenix (Becton Dickinson, USA), disc zone diameter or gradient diffusion methods (e.g. E-test, BioMerieux, France) [3]. Genotypic susceptibility testing, or the detection of genes associated with antimicrobial resistance [3], such as *van* genes, is utilized in some settings.

The aim of this study was to assess the frequency of ‘missed’ VRE by performing genotypic testing on *E. faecium* isolates from sterile body sites categorized as vancomycin susceptible by phenotypic susceptibility testing. Secondly, we wanted to consider the clinical significance of these ‘silent’ genes if detected, particularly in the context of accessibility and turnaround time of currently available commercial genotypic assays.

## Methods

A retrospective audit of vancomycin-susceptible *E. faecium* (VSE) isolates was carried out. VSE isolated from sterile site specimens (blood, fluid, tissue and CSF) from June 2020 to June 2022 were included. The isolates had all been processed at our tertiary hospital laboratory and may have originated from any location within our health district. The isolates had been categorized as phenotypically vancomycin susceptible by VITEK2 (BioMerieux, France) per standard operating procedure in our laboratory. Each isolate underwent genotypic testing using the Xpert vanA/vanB (Cepheid, Sunnyvale, USA): a rapid, real-time Nucleic Acid Amplification Test (NAAT) for the detection of *vanA* and *vanB* in rectal screening swabs. Because the assay is validated by Cepheid for rectal swabs only, the method for detection from colonies was derived from experimental methods that have been used by other laboratories as described in personal communication with Cepheid. Briefly, 75 µl of a 1:1000 dilution of a 0.5 McFarland solution was added to the GeneXpert test cartridge, and the results were interpreted by the GeneXpert System with a run time of approximately 40 min. Quality control for this method was performed using kit and well-characterized clinical and external quality assurance programme controls. Results were confirmed using the AusDiagnostics Staphylococcus and VRE eight-well assay, validated for use on culture isolates (AusDiagnostics, Australia). Advice was sought from the NSW Health Pathology Research Governance office who waived the requirement for review by Human Research Ethics Committee due to the negligible ethical risks.

## Results

A total of 50 clinical isolates meeting criteria were retrieved from freezer storage. Twenty-three (46%) were from blood cultures, 8 (16%) were from tissue and 19 (38%) were from sterile fluids. Three out of 50 isolates [6%; 95% CI 1.3–16.5% (Fisher Exact method)] had a *vanA* gene detected by the Xpert VRE assay. Two of these were isolated from blood, and the other was isolated from tissue. All three had tested susceptible to vancomycin and teicoplanin with MICs of <0.5 mg l^−1^ by VITEK2 prior to storage. After revival from storage, vancomycin susceptibility was confirmed by gradient diffusion (E-test, BioMerieux, France) for each isolate with MICs of 1–1.5 mg l^−1^, followed by subculture onto Brilliance VRE chromogenic screening agar (ThermoFisher, USA), with no growth occurring on this media. All 50 isolates, including the three *vanA-*positive isolates, were referred for testing on the AusDiagnostics Staphylococcus and VRE multiplex assay, validated for use on culture isolates, with 100% concordance. All three *vanA* detections were from patients with urosepsis who did not have documented clinical failure with vancomycin therapy; however, one patient was palliated within 6 months due to progressive osteomyelitis, for which it remained unclear whether the *E. faecium* was a contributing cause, and another patient later isolated a phenotypic *vanA* isolate from a screening swab.

## Discussion

A total of 6% (95% CI 1.3–16.5%) of isolates that were determined to be vancomycin susceptible by phenotypic method were found to carry vancomycin resistance genes by the Xpert VRE assay. The significance of these results depends on various factors, including whether the presence of *van* genes is sufficient to determine clinical vancomycin resistance, the clinical and systemic impacts of misidentifying VRE as VSE and the feasibility and cost of genotypic assays.

In recent years, it has been noted that some VRE, particularly *vanB-*carrying isolates, can evade detection on phenotypic vancomycin susceptibility testing [4,5]. In 2018, the European Committee on Antimicrobial Susceptibility Testing (EUCAST) issued a warning against the use of vancomycin gradient tests alone to detect low-level vancomycin resistance. It was also suggested that if disc diffusion was being used, one should carefully observe for any fuzziness along the zone edge and that if there was any uncertainty regarding interpretation, then genotypic testing should be used for confirmation [4]. This is of particular relevance for *vanB*-expressing isolates as they are more likely to display low-level vancomycin resistance, closer to breakpoints, and importantly, these MICs can increase with vancomycin exposure [5]. Additionally, the phenomenon of vancomycin-variable *Enterococci* (VVE), which harbour *van* genes but do not display phenotypic resistance until exposed to glycopeptide antibiotics, has been described [5]. The VSE isolates with *vanA* genes detected in our study would likely fall into this VVE category.

The exact mechanism for the VVE phenotype remains unclear. It is postulated that the *vanA* gene is susceptible to insertion sequence-mediated alterations that can affect the expression of resistance and result in a *vanB* or even vancomycin-sensitive appearing phenotype on susceptibility testing. In 2014, a Canadian report described a cluster of clinical failures with infections caused by phenotypic VSE when treated with vancomycin; the isolates were found to be carriers of *vanA* [6]. After exposure to glycopeptide and re-isolation, subsequent phenotypic testing was consistent with VRE, with genotypic assays confirming the presence of *vanA* genes [6]. A Norwegian study in 2016 had similar observations, with whole-genome sequencing detecting a silencing insertion sequence (IS*L3*) proximal to the *vanHAX* region that was selected out after exposure to vancomycin, allowing the expression of the *van* gene [7]. The estimated proportion of VSE isolates carrying silent *van* genes varies by study, with 7.1% reported in a 2024 Korean-based study and 3.6% in the 2019 Australian data, and *vanA* is most commonly seen [8,9]. While still uncommon, or at least uncommonly recognized, outbreaks of VVE have been seen in several countries including a significant and prolonged outbreak in Denmark, as well as Korea, India and Italy [10]. Genotypic *van* testing for invasive VSE isolates has been recommended by sites involved in outbreak investigation [6], and some centres have subsequently adopted routine genotypic screening methods.

It is essential to consider the clinical significance of VRE (or potential VRE) and, therefore, the significance of misidentifying it. Even with appropriate targeted treatment, clinical infection with VRE is associated with significant morbidity and mortality and is increasing in prevalence [11]. While estimates vary, VRE bloodstream infection is thought to have as much as a 2–3× increased mortality compared with VSE [11]. *In vitro*, >90% of VVE have been found to revert to a vancomycin-resistant phenotype with exposure to vancomycin [6,12]. However, the frequency and factors associated with clinical conversion of VVE to VRE remain unknown. While there are anecdotal case reports, there is no sufficient clinical evidence to confirm that treatment failures are solely attributable to conversion of VVE to VRE during vancomycin therapy. Additionally, neither EUCAST nor the Clinical & Laboratory Standards Institute (CLSI) currently recommend routine confirmatory genotypic testing for *E. faecium*. To compare with similar situations, the detection of ‘silent’ genes is seen in other organisms. For example, Carvalho *et al*. [13] reported that 16% of *Acinetobacter baumannii* strains harboured silent *bla_OXA-23_*. Similarly, a silent *mcr-1* gene was reported in *Escherichia coli* in 2016 [14]. However, antimicrobial society guidelines such as EUCAST and CLSI recommend interpreting susceptibility for third-generation cephalosporins for Enterobacterales based on clinical breakpoints and do not recommend genotypic confirmation for purposes other than infection control reasons. The same analogy applies to VVE wherein reporting vancomycin based on phenotypic susceptibility, as opposed to genotypic testing, may be considered.

Lastly, it is important to consider that the prevalence of vancomycin-susceptible *E. faecium* in blood cultures (and other sterile sites of clinical significance) is relatively low. For example, at our public hospital laboratory covering a population of more than 840,000, there were only 13 unique VSE isolations from blood cultures in 2022, and as discussed, only a small proportion of these isolates harbour silent *van* genes. For a laboratory, the workflow impact of an additional genotypic test on VSE, therefore, comes with minimal additional cost and manual labour. However, this also calls for an assay on a ‘random-access’, as opposed to a batched, platform. At the time of writing, there were very few commercial NAAT assays available to detect the presence of *van* genes from culture isolates. Consequently, many laboratories relied on batched assays, and results were often not available in a clinically useful timeframe.

There are limitations to this study. The sample size was small. The *vanA*/*vanB* GeneXpert has a sensitivity of 96% (95% CI 93–98%) and specificity of 90% (95% CI 88–91%) [12]; however, this is validated by Cepheid for use on swabs only, not colonies. The colony method described has been used only experimentally by other laboratories, though it underwent internal validation using both negative and positive colony controls in our laboratory, as well as external validation with 100% concordance on the AusDiagnostics assay. Additionally, while *vanA*/*vanB* are the most common genes conferring vancomycin resistance, rarely other genes can be responsible, for example, *vanC*, *vanD*, *vanE* and *vanG*, which would not be detected in this assay [1].

## Conclusion

A total of 6% of sterile site VSE isolates were found to carry vancomycin resistance genes. However, while genotypic testing of VSE may indicate the presence of putative resistance genes missed on routine susceptibility testing, further research is required to determine its diagnostic relevance and to validate the method used in this study.

## Abbreviations

CLSI: Clinical & Laboratory Standards Institute
EUCAST: European Committee on Antimicrobial Susceptibility Testing
NAAT: nucleic acid amplification test
VRE: vancomycin-resistant *Enterococci*
VSE: vancomycin-susceptible *Enterococci*
VVE: vancomycin-variable *Enterococc*i
